# Dr. Robert Battey

**Published:** 1895-10

**Authors:** 


### *EDITORIAL.*

---

The office of THE JOURNAL is in room 330 Equitable Building.

The editors are not responsible for views expressed by contributors.

Articles for publication should reach this office not later than the 15th instant.

Address all communications, and make all remittances payable to THE ATLANTA MEDICAL AND SURGICAL JOURNAL, Atlanta, Ga.

Reprints of articles will be furnished, when desired, at a nominal cost. Requests for the same should always be made *on the manuscript*.

---

### DR. ROBERT BATTEY.

As we go to press the papers announce the serious illness of Dr Battey of Rome, and it appears that his death may be expected at any moment. To the medical profession of the entire country this announcement will be read with extreme regret.

Dr. Battey's name and work are known wherever scientific medicine is practiced and medical literature read. Dr. Battey has enjoyed a highly successful professional career. After graduating from the Jefferson Medical College in 1857, he located in Rome where the greater part of his life has been spent. During the war he was commissioned surgeon in the Confederate army, and in this capacity served with great ability in the hospitals of Rome, Atlanta, Macon, and other places. The most notable feature of his professional life was his resort to ovariotomy "with a view to establish at once the change of life for the effectual remedy of certain otherwise incurable maladies." His first operation was done August 17, 1872. It was the first ovariotomy done by a Georgia surgeon. Dr. Battey was first bitterly criticised, and then cordially indorsed. In November, 1872, Dr. Battey moved to Atlanta, and become Professor of Obstetrics and Gynecology in the Atlanta Medical College, and filled this chair with distinction for three years. During the same time he was editor of THE ATLANTA MEDICAL AND SURGICAL JOURNAL. He returned to Rome in 1875 and erected his Infirmary for diseases of women, which has continued in successful operation to this time. In 1876 Dr. Battey was elected President of the Georgia Medical Association, and in 1889 he was made President of the American Gynecological Society. He contributed many important papers to medical literature; these have been published for the most part in THE ATLANTA MEDICAL AND SURGICAL JOURNAL, the *Virginia Medical Monthly*, the *Transactions of the Georgia Medical Association*, the *Transactions of the American Gynecological Society*, and the *British Medical Journal*.

Personally Dr. Battey is a gentleman of the highest character whom no one speaks of save with love and esteem. He has possessed to a high degree the confidence of patients and professional associates, and his death will be genuinely mourned in many homes.

A more extended notice will appear in another issue.

